# Systematic review: locating qualitative academic publications for reviewing tenants’ and landlords’ renting experiences and interaction in the Majority World

**DOI:** 10.12688/openreseurope.18234.1

**Published:** 2024-08-13

**Authors:** Adriana Mihaela Soaita

**Affiliations:** 1 Sociology, University of Bucharest, Schitu Magureanu 9 Bucharest, 030167, Romania; 2 Urban Studies, University of Glasgow, 25-29 Bute Gardens Glasgow, Scotland, G12 8RS, UK

**Keywords:** Systematic review, Methodological protocol, Tenants, Landlords, Private renting, Private rental housing, Majority World, Literature mapping, SCOPUS, Web of Science

## Abstract

This methodological protocol describes the step-by-step process of identifying the relevant international academic literature to be reviewed within the project ‘The affective economies of emerging private renting markets: understanding tenants and landlords in postcommunist Romania” (AFFECTIVE-PRS). It presents: (1) the preliminary decisions taken related to the breadth of the review (choice of databases, type of research, type of reference, searching fields); (2) the operationalisation of keywords and Boolean strings; (3) the further calibration of the searching parameters through piloting; (4) the final retrieval of relevant references through systematic and manual searches; and (5) the geographical coverage of the retained literature. While the paper demonstrates the rigour of the methodological approach taken, it also opens up the space for other scholars to scrutinise, replicate or adjust this approach to their own work.

## Introduction

The 2008–2010 Global Financial Crisis and the Covid-19 pandemic have highlighted rental housing as a significant source of inequalities in wealth, health, and well-being worldwide, often failing to provide many tenants with a true sense of 'home.' A burgeoning recent academic literature focusing on Anglo-Saxon countries and older EU member states has revealed troubling issues, such as poor housing quality, insecurity, eviction experiences and anxieties, and economic stress. These findings raise valid concerns about tenants' well-being in the less institutionalised and therefore riskier private renting sectors (PRS), where informal transactions elevate risks and conceal vulnerabilities from state action.

The project titled “The Affective Economies of Emerging Private Renting Markets: Understanding Tenants and Landlords in Postcommunist Romania (AFFECTIVE-PRS)” addresses these important concerns. The project aims to explore the affective economies that connect private tenants and landlords in the understudied regions of the “Majority World” ( Simone & Fauzan, 2013) that is the less developed countries of the Global South and Global East. To achieve this, the project will develop a Critical Interpretative Synthesis (CIS) of relevant academic literature. CIS is a novel review approach ( Depraetere *et al.*, 2021) described as 'a methodology that enables the synthesis of large amounts of diverse qualitative data and facilitates critical engagement with the assumptions that shape and inform a body of research' ( Farias & Laliberte Rudman, 2016 p:33).

Several systematic and scoping reviews have focused on the everyday experiences of private tenants in the developed states of the “Minority World,” particularly in the well-researched Anglo-Saxon countries ( Chisholm *et al.*, 2020; Garnham & Rolfe, 2019; McCarthy & Simcock, 2024; Rolfe *et al.*, 2023; Soaita *et al.*, 2020; Soaita *et al.*, 2022; Woodhall-Melnik & Dunn, 2016). In contrast, only one scoping review on rental arrangements in the Majority World has been identified, and it dates back nearly 30 years ( Rakodi, 1995). Considering that the challenges faced by private tenants and landlords in the Majority World are likely to be more salient and fundamentally different from those elsewhere, assembling this expectedly small and fragmented literature for synthesis holds significant academic and ethical importance for knowledge representation.

Building on previous efforts to develop a feasible approach within limited resources ( Soaita *et al.*, 2019b; Soaita *et al.*, 2020; Soaita *et al.*, 2023), a Critical Interpretative Synthesis (CIS) will address the following questions:

1. What is the geographical and temporal distribution of academic knowledge concerning the affective and everyday experiences and interactions between private tenants and landlords in the Majority World?
2. What are the collective affective experiences that drive the actions and interactions of private tenants and landlords in the Majority World?

This methodological protocol addresses the first question through literature mapping ( Soaita *et al.*, 2019b), while the second question will be explored elsewhere by thematically synthesising a subset of the sampled literature. However, the primary contribution of this protocol is methodological, as it presents a rigorous approach, which is also feasible within the limited resources typically available to academic projects, in contrast to the resource-rich traditional systematic reviews. Additionally, it enhances the AFFECTIVE-PRS project’s transparency by making the full set of references FAIR—Findable, Accessible, Interoperable, and Reusable—for other academic users. The paper not only demonstrates the rigour of the adopted methodological approach but also invites other scholars to scrutinise, replicate, or adapt it for their own research. By focusing on the retrieval of relevant literature rather than its synthesis, this paper aims to assist and inspire scholars and students across all social science disciplines.

The paper advances by detailing the following aspects:

- Preliminary decisions regarding the scope of the review
- The operationalisation of Boolean search strings
- The calibration of search parameters through thorough piloting
- The final retrieval of relevant publications via both systematic and manual searches
- A step-by-step summary of the process, including a geographical mapping of the results

It concludes with a brief summary.

## Preliminary decisions

To align with the AFFECTIVE-PRS resources and ensure sufficient bibliographical breadth in retrieving high-quality academic publications, several restrictive decisions were made from the outset. These decisions will inevitably result in some relevant references being missed during the systematic searches. However, to mitigate this and as commonly recommended, the systematic searches will be complemented by manual searches in a select number of key journals and by recommendations from peers.

### Databases

To systematically review the published literature on tenants’ and landlords’ experiences of renting, the extensive and complementary databases SCOPUS and Web of Science were selected. This approach aims to ensure broad coverage while acknowledging that the review will not be exhaustive ( Soaita *et al.*, 2019b).

### Search fields

Based on prior experience ( Soaita *et al.*, 2019a; Soaita *et al.*, 2019b; Soaita *et al.*, 2023), searches will be performed in the fields of ‘title’, ‘abstract’ and ‘keywords’ in SCOPUS and ‘topic’ in Web of Science in order to get the most relevant results.

### Type of research

Given the qualitative nature of the AFFECTIVE-PRS’s research questions, the decision was made to focus exclusively on qualitative studies. This approach builds on Soaita *et al.* (2020), expanding their scope from Anglo-Saxon countries to the Majority World.

### Type of references

Following others ( Lee *et al.*, 2015; Wang *et al.*, 2016), as a way to proxy research quality, the decision was made to include only journal articles, books and book chapters.

## Operationalisation of the search strings

 Drawing on Cooke *et al.* (2012) and the PICO method, the three search strings were designed to capture:

- Population and Comparison terms: tenants and/vs. landlords;
- Intervention: the field of housing (rather than other rental markets, e.g. land, commercial property);
- Outcomes: well/ill-being, through the proxy of methodology (i.e. qualitative research).

The operationalisation of each search string was designed to be inclusive of key meta-categories (e.g., housing, qualitative) and not to miss specific subcategories (e.g., flatmate, rooming house). This approach aimed to ensure that, collectively, the search strings would return the most relevant publications. Table 1 details the focus and operationalisation of the three search strings, which will be used together. An asterisk (*) is used to include both the singular and plural forms of a keyword.

**Table 1.** The search strings.

Strings	Fields	Operationalisation
String 1	Tenants OR landlords	(private AND (tenant* OR renter*)) OR lodger* OR squatter* OR flatmate* OR (HMO* AND resident*) OR (HMO* AND tenant*) OR (private AND landlord*) OR (tenant* AND landlord*) OR (landlord*-tenant* OR tenant*-landlord*)
String 2	Housing	(housing OR home OR house OR flat OR apartment OR flat-share OR (hous* AND “in multiple occupation”) OR (rooming AND hous*) OR (boarding AND hous*) OR (Rooming AND hous*))
String 3	Method	(qualitative OR interview OR ethnograph* OR “case study” OR “case studies”)

However, any search string must be piloted to understand the characteristics of the publications so retrieved. Given its analytical tools, SCOPUS is particularly useful for preliminary piloting.

## Calibration of search parameters: a SCOPUS pilot

### Piloting the keywords

The keywords for String 1 (see Table 1) were piloted separately in SCOPUS on January 9, 2023. This initial test revealed that certain keywords were heavily associated with specific disciplines (e.g., "flat*" and "flatmate*" returned many results in the physical sciences, and "boarding-hous*" in biology and veterinary sciences). However, these disciplinary associations diminished when Strings 2 and 3 were added, effectively refining the search results to better target the relevant literature.

It was also observed that introducing the housing String 2 reduced the total hits by approximately 13% for “private renter”, 30% for “private landlord” and 49% for “tenant and landlord”. This indicates that papers focused on renting other assets (e.g. land, money, commercial space) and seldom included both tenants and landlords.

Further refining the results with String 3 led to a substantial reduction in housing-related hits by approximately 70%, highlighting the predominantly quantitative nature of most publications returned.

Given AFFECTIVE-PRS's interest in regions beyond Anglo-Saxon geographies, the volume of literature related to Australia, New Zealand, the UK, and the USA was carefully monitored. Similar to findings in other studies ( Liu *et al.*, 2021; Soaita *et al.*, 2023), the piloting exercise revealed that, for all keyword combinations, 50% to 80% of the hits pertained to these four Anglo-Saxon countries in terms of the geography of the case studies.

### Setting additional inclusion/exclusion criteria

The SCOPUS literature was subsequently retrieved on January 10, 2023, using the search query “String 1 AND String 2 AND String 3”. This search returned a total of 552 references of articles, book and book chapters. Upon examining the characteristics of these references, further exclusion decisions were made based on specific criteria:

- Discipline: 35 references were excluded ^1^
- Language: 13 were excluded for being written in a language other than English
- Publishing timeline: given that a systematic review of the pre-1992 rental housing literature was already available ( Rakodi, 1995) and only 12 of the retrieved references were published between 1973 and 1989, it was decided to focus the review exclusively on studies published after 1990.

Overall, it was determined that the combination of the three search strings, along with the additional excluding criteria, would yield a body of literature that is sufficiently focused on the topic while providing ample geographical, substantive and theoretical diversity.

### The Majority World

**Figure 1.**
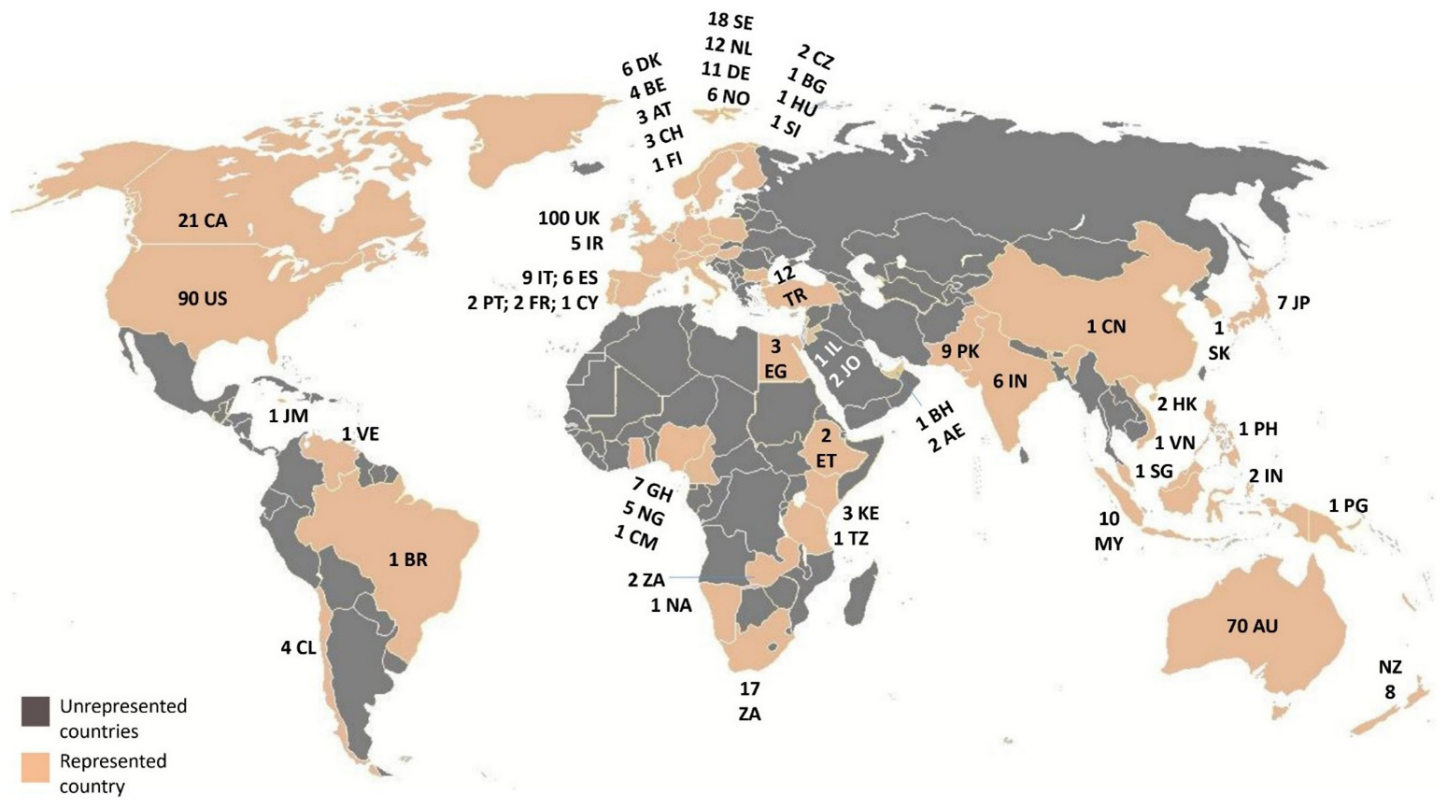
The global spread of the (SCOPUS) retrieved literature.

The remaining sample (n=492) was downloaded in an EndNote database, where it was coded for countries of the case studies, then mapped in order to assess geographical coverage. As noted earlier, the results revealed a predominant focus on Anglo-Saxon literature (and some western European countries) in the geographical distribution of the case-studies (see Figure 1). Additionally, this dominance extended to institutional affiliation, authorship, and funding sources, as detailed in Soaita (2023) ( https://zenodo.org/records/7566096).

Figure 1 highlights the lack of representation of many Majority World countries, particularly in Eurasia and Africa, which are marked in grey. Some regions, like Brazil, are represented by only a single paper. Although Liu *et al.* (2021) observed that China was well represented in the economic housing literature, ranking second after the USA, this was not the case in this pilot exercise.

To focus on the emerging and less-studied private renting markets in the Majority World ( Simone & Fauzan, 2013), literature from Anglo-Saxon countries, old EU members and some other developed states would be obviously excluded. ^2^ While informal renting arrangements do exist in these regions, they are relatively marginal ( Morris *et al.*, 2021). The developed world accounted for 378 references, or 77% of the retrieved sample, highlighting the substantial under-representation of the Majority World in academic literature. Consistent with Kholodilin (2020), the decision was made to include all Majority World states in the final review, notwithstanding their diverse renting contexts and uneven and fragmented representation.

## Retrieving the literature for reviewing

### Systematic searches

Once the exclusion/inclusion criteria have been calibrated to the study’s aim, the literature was retrieved on January 10 2023 through systematic searches in SCOPUS and Web of Science (90 and 64 references respectively, both imported into EndNote). In more detail ^3^:

- **SCOPUS Boolean string**: (TITLE-ABS-KEY (( **private** AND ( tenant* OR renter*)) OR lodger* OR squatter* OR flatmate* OR ( hmo* AND resident*) OR ( hmo* AND tenant*) OR ( **private** AND landlord*) OR ( tenant* AND landlord*) OR ( landlord*-tenant* OR tenant*-landlord*)) AND TITLE-ABS-KEY (( **housing** OR **home** OR **house** OR **flat** OR **apartment** OR **flat-share** OR ( hous* AND **"in multiple occupation"**) OR ( **rooming** AND hous*) OR ( **boarding** AND hous*))) AND TITLE-ABS-KEY (( **qualitative** OR **interview** OR ethnograph* OR **"case study"** OR **"case studies"**))) AND (EXCLUDE (AFFILCOUNTRY, **"United Kingdom"**) OR EXCLUDE (AFFILCOUNTRY, **"United States"**) OR EXCLUDE (AFFILCOUNTRY, **"Australia"**) OR EXCLUDE (AFFILCOUNTRY, **"Canada"**) OR EXCLUDE (AFFILCOUNTRY, **"Sweden"**) OR EXCLUDE (AFFILCOUNTRY, **"Germany"**) OR EXCLUDE (AFFILCOUNTRY, **"Netherlands"**) OR EXCLUDE (AFFILCOUNTRY, **"Italy"**) OR EXCLUDE (AFFILCOUNTRY, **"Japan"**) OR EXCLUDE (AFFILCOUNTRY, **"New Zealand"**) OR EXCLUDE (AFFILCOUNTRY, **"Denmark"**) OR EXCLUDE (AFFILCOUNTRY, **"Ireland"**) OR EXCLUDE (AFFILCOUNTRY, **"Norway"**) OR EXCLUDE (AFFILCOUNTRY, **"Spain"**) OR EXCLUDE (AFFILCOUNTRY, **"Austria"**) OR EXCLUDE (AFFILCOUNTRY, **"Belgium"**) OR EXCLUDE (AFFILCOUNTRY, **"France"**) OR EXCLUDE (AFFILCOUNTRY, **"Switzerland"**) OR EXCLUDE (AFFILCOUNTRY, **"Portugal"**) OR EXCLUDE (AFFILCOUNTRY, **"Cyprus"**) OR EXCLUDE (AFFILCOUNTRY, **"Finland"**)) AND (LIMIT-TO (DOCTYPE, **"ar"**) OR LIMIT-TO (DOCTYPE, **"ch"**) OR LIMIT-TO (DOCTYPE, **"re"**) OR LIMIT-TO (DOCTYPE, **"bk"**)) AND (EXCLUDE (PUBYEAR, **1989**) OR EXCLUDE (PUBYEAR, **1988**) OR EXCLUDE (PUBYEAR, **1987**) OR EXCLUDE (PUBYEAR, **1986**) OR EXCLUDE (PUBYEAR, **1985**) OR EXCLUDE (PUBYEAR, **1984**) OR EXCLUDE (PUBYEAR, **1982**) OR EXCLUDE (PUBYEAR, **1976**)) AND (LIMIT-TO (LANGUAGE, **"English"**)) AND (EXCLUDE (SUBJAREA, **"MEDI"**) OR EXCLUDE (SUBJAREA, **"EART"**) OR EXCLUDE (SUBJAREA, **"AGRI"**) OR EXCLUDE (SUBJAREA, **"BIOC"**))
- **Web of Science Boolean string**: (private AND (tenant* OR renter*)) OR lodger* OR squatter* OR flatmate* OR (HMO* AND resident*) OR (HMO* AND tenant*) OR (private AND landlord*) OR (tenant* AND landlord*) OR (landlord*-tenant* OR tenant*-landlord*) (Topic) AND (housing OR home OR house OR flat OR apartment OR flat-share OR (hous* AND “in multiple occupation”) OR (rooming AND hous*) OR (boarding AND hous*)) (Topic) AND (qualitative OR interview OR ethnograph* OR “case study” OR “case studies”) (Topic) and Infectious Diseases or Forestry or Water Resources or Genetics Heredity or Mathematical Computational Biology or Archaeology or Biochemistry Molecular Biology or Biotechnology Applied Microbiology or Chemistry or Critical Care Medicine or Emergency Medicine or Imaging Science Photographic Technology or Instruments Instrumentation or Mechanics or Medical Ethics or Neurosciences Neurology or Oceanography or Parasitology or Physics or Polymer Science or Food Science Technology or Meteorology Atmospheric Sciences or Pharmacology Pharmacy or Plant Sciences or Zoology or Agriculture or Geriatrics Gerontology or Veterinary Sciences or Surgery or Mathematics or Nutrition Dietetics or Geology or Immunology or Obstetrics Gynecology or Gastroenterology Hepatology (Exclude – Research Areas) and English (Languages) and ENGLAND or USA or UNITED KINGDOM or AUSTRALIA or CANADA or SCOTLAND or SWEDEN or NETHERLANDS or GERMANY or ITALY or NEW ZEALAND or NORWAY or DENMARK or FRANCE or NORTH IRELAND or SPAIN or BELGIUM or IRELAND or FINLAND or GREECE or UK or AUSTRIA or or JAPAN or PORTUGAL or SWITZERLAND or WALES (Exclude – Countries/Regions)

Notwithstanding the precision of the search strings and the automatic removal of duplicate references by EndNote (n=34), the retrieved literature must always be manually assessed for relevance. Of the 120 unique references retrieved, only 27 were retained for reviewing. Although there is no associated data, to ensure FAIR reporting of the process, Box 1 under Source Data lists all excluded references by type of misfit (e.g. language, country, thematic/method), with a few being inaccessible or previously undiscovered duplicates. The largest group of exclusions (n=66) was due to thematic or methodological mismatches, their focus being on public/social renting, general housing or renting policies, homeownership while others employed quantitative methods or were tenure-blind in their discussion. However, some of these references were set aside for a better understanding of the policy context.

### Manual searches

Best practice ( Arksey & O'Malley, 2005; Barnett-Page & Thomas, 2009; Carroll *et al.*, 2011; Cooke *et al.*, 2012; Erasmus *et al.*, 2014; Evans & Benefield, 2001) advocates for enhancing the systematically retrieved sample by incorporating manual searches in key journals, seeking peer recommendations, and reviewing the reference lists of the identified publications.

As the 27 references were dispersed across a wide range of journals, with none featuring more than twice, specific journals were selected for further manual searches: *Housing Studies* and the *International Journal of Housing Policy* for their international focus on housing; *Geoforum* and *Urban Studies* for their international albeit broader substantive focus; and *Habitat International* for its emphasis on developing countries. *Google Scholar* was also used. Searches were conducted between January 12 and 16, 2023. As a result, 35 additional references were incorporated, bringing the total to 62. Peer recommendations produced no additions to the sample, which reinforces confidence in the relevance of the sample.

Based on previous work ( Soaita, 2018a; Soaita, 2018b; Soaita *et al.*, 2019a; Soaita *et al.*, 2019b; Soaita *et al.*, 2020; Soaita *et al.*, 2023; Soaita *et al.*, 2022), it was considered that a sample of 62 publications is sufficiently large to provide both theoretical and geographical nuance while remaining manageable within the project's time and resource constraints.

### Further additions

During the analysis phase, authors will retain the flexibility to add and review new references if they are deemed particularly relevant, especially by checking the reference lists of the retrieved publications. Given the very fragmented geographical distribution of the retrieved literature, the decision was made to focus the analysis on three key regions: Western Africa and the Indian subcontinent, which were relatively well-represented, and Eastern Europe, which is highly relevant to the AFFECTIVE-PRS project’s empirical case study of Romania. As a result, 15 additional publications were incorporated based on reference checks and peer recommendations. Consequently, the final sample of retrieved literature comprises 77 publications.

## Summary and mapping

Table 2 summarises the process, showing the steps, the action, the number of references at the end of each action, and explanatory notes. A detailed PRISMA checklist and a flow diagram can be consulted in Soaita (2024) ( https://zenodo.org/records/12794284).

**Table 2.** Summary of the process.

Steps	Action	No.	Explanatory notes
S1	Preliminary steps		Setting and piloting the parameters of the systematic searches (e.g. choice of databases, searching fields, type of reference, discipline, publishing timeline, geography).
S2	Retrieved	154	Systematic searched in SCOPUS and Web of Science (January 10, 2023). References exported in an EndNote database.
S3	Unique references	120	EndNote automatic removal of 34 duplicates.
S4	Manual check for relevance (in titles, abstracts and full text)	27	93 references excluded due to: language misfit (n=1), inaccessibility (n=6), unrecognised duplicates (n=7), country misfit (n=13) and thematic or methodological misfit (n=66).
S5	Boosting the sample with manual searches	62	35 new references were added following searches conducted between January 17 and 20, 2023. (Housing Studies, International Journal of Housing Policy, Geoforum, Habitat International, Urban Studies), and Google Scholar.
S6	Reviewing the reference lists of selected retrieved publications	77	15 references were added after the decision was made to focus the analysis on the regions of Eastern Europe, Western Africa and the Indian sub-continent.

Note: * The final samples at stage S4 and S5 were refined during stage S6, differing slightly from that reported in in Soaita (2023) ( https://zenodo.org/records/7566096).

**Figure 2.**
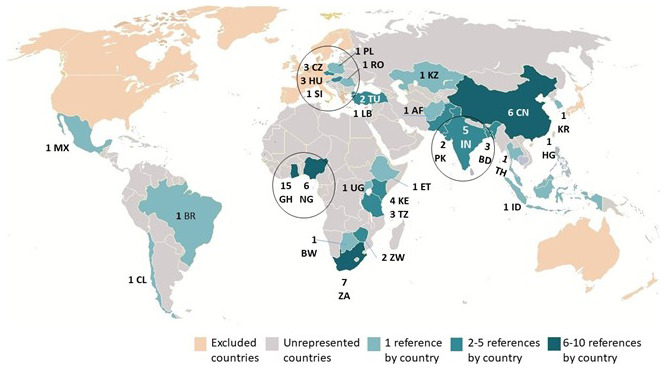
The geographical distribution of the final sample of publications.

Figure 2 displays the geographical distribution of the 77 publications. It addresses the first research question about the geographical scope of academic knowledge concerning private tenants’ and landlords’ renting experiences and interaction in the Majority World. The figure reveals several key points: many countries remained unrepresented in the sample. Of the 29 represented countries: 16 countries are included by only one publication each; eight countries have between 2 and 5 papers; and only four countries have more than 5 papers. These observations underscore the need for further research into the renting arrangements and experiences in the Majority World.

The publishing timeline of the 77 references retained for review reveals that the majority were published after 2008, accounting for 70% of the total (n=54). In addition to these 77 publications, some papers were retained to provide broader context on housing policies in the Global South, including two systematic reviews of rental tenure ( Gilbert, 2016; Rakodi, 1995). ^4^ Although there is no associated data, to ensure the reviewing approach is FAIR, Box 2 under Source Data categorises all included references by search method (i.e. systematic, manual and additional).

## Conclusion

This methodological protocol aims to enhance the AFFECTIVE-PRS reviewing approach by making it FAIR—Findable, Accessible, Interoperable, and Reusable for other academic users. It outlines the rigorous methodology employed to identify the relevant publications for review, while adapting to the project’s limited time and human resources. The protocol also provides a foundation for other scholars to scrutinise, replicate, or adjust this approach for their own studies. It details the preliminary decisions that defined the review’s scope; the operationalisation of Boolean strings for systematic searches; the calibration of parameters through thorough piloting; and the retrieval of the final sample of publications. Additionally, it offers a step-by-step summary of the process and geographical mapping of the results. Findings, including coding, text extraction and data analysis will be presented in a separate paper.

## Source data

Although there is no data associated with this methodological protocol, Box 1 and Box 2 list all references in order to ensuring transparency and accessibility of the review process according to the FAIR principles. Box 1 lists the 93 excluded references categorised by type of misfit, while Box 2 provides details of the 77 references retained for review, organised by search method (systematic, manual, and additional).

**Box 1. Excluded references by category of misfit**

**Table:** 

Language misfit (n=1)
1. Pérez, M., and Palma, C. (2021). "From foreigners to urban citizens: autoconstruction and migration in the Santiago Metropolitan Area." *Estudios Atacamenos*, 67, 1–21.
Not accessible (n=6)
2. Adianto, J., Gabe, R. T., and Sihombing, A. (2022). "Houses with permeable walls: a case study from Kampong Kwitang, Central Jakarta." *International Journal of Design in Society*, 16(2), 1–18. 3. Bocutoğlu, E., and Ertürk, Z. (2006). "Supply and demand analysis in the housing market: a case study in Turkey as a developing country", *Management, Quality and Economics in Building*. CRC Press, pp. 1566–1572. 4. Brezar, V. (2012). "The future of urban dwelling design." *International Journal for Housing Science and Its Applications*, 36(2), 89–98. 5. Kamruzzaman, M. (2012). *Housing for the urban poor through informal providers, Dhaka, Bangladesh*. 6. Kayila, J. O. (2019). "Improving urban settlements for the poor: case studies of Dandora and Chaani projects in Kenya", *Reaching the Urban Poor: Project Implementation in Developing Countries*. Taylor and Francis, pp. 145–162. 7. Lee-Smith, D. (2018). "Squatter landlords in Nairobi: a case study of Korogocho", *Housing Africa'S Urban Poor: Volume 2*. Taylor and Francis, pp. 175–188.
Unrecognised duplicates (n=7)
8. Akhtar, A. S., and Rashid, A. (2021). "Dispossession and the militarised developer state: financialisation and class power on the agrarian-urban frontier of Islamabad, Pakistan." *Third World Quarterly*, 42(8), 1866–1884. 9. Govender, V., and Loggia, C. (2021). "Adaptive reuse strategies in Durban inner city using hybrid mapping tools" *Urban Book Series*. Springer Science and Business Media Deutschland GmbH, pp. 219–250. 10. Govindasamy, A. R. (2010). "Indians and rural displacement: exclusion from region building in Malaysia." *Asian Journal of Political Science*, 18(1), 90–104. 11. Jackson, A., and Archer, C. D. (2022). "Factors influencing Jamaican householders’ housing choice." *International Journal of Housing Markets and Analysis*, 15(5), 1053–1071. 12. Kotyk, L. (2020). "Governing without governed and governors: an attempt to establish a non-hierarchical organizational repertoire1." *Partecipazione e Conflitto*, 13(3), 1308–1323. 13. McKay, T., Fakudze, N., and Gunter, A. (2022). "The middle remains missing: class exclusion from the urban rental market in suburban Johannesburg." *Acta Academica*, 54(1), 113–133. 14. Yung, B., and Lee, F. P. (2014). "‘Equal right to housing’ in Hong Kong housing policy: perspectives from disadvantaged groups." *Journal of Housing and the Built Environment*, 29(4), 563–582.
Country misfit (n=13)
15. Bennett, J., Howden-Chapman, P., Chisholm, E., Keall, M., and Baker, M. G. (2016). "Towards an agreed quality standard for rental housing: field testing of a New Zealand housing WOF tool." *Australian and New Zealand journal of public health*, 40(5), 405–411. 16. Cox, G., and Phibbs, P. (1994). "Evaluation of a tenancy advice service case study of Waverley, Sydney." *Urban Policy and Research*, 12(1), 40–46. 17. Dorn, V. (1997). "Changes in the social rented sector in Germany." *Housing Studies*, 12(4), 463–475. 18. Kadir, N. (2016). *The Autonomous Life? Paradoxes of Hierarchy and Authority in the Squatters Movement in Amsterdam*: Manchester University Press. 19. Kotyk, L. (2020). "Governing without governed and governors: an attempt to establish a non-hierarchical organizational repertoire1." *Partecipazione e Conflitto*, 13(3), 1308–1323. 20. Sim, D. (1996). "The Scottish house factoring profession." *Urban History*, 23(3), 351–370. 21. Smith, M. P. (2017). *Marginal Spaces: Comparative Urban and Community Research,* volume 5: Taylor and Francis. 22. Stanbury, W. T., and Todd, J. D. (1990). "Landlords as economic oners of war." *Canadian Public Policy/Analyse de Politiques*, 16(4), 399–417. 23. Vanderwood, P. J. (2003). *Night riders of Reelfoot lake*: University of Alabama Press. 24. Varady, D. P., Walker, C. C., McClure, K., Smith-Heimer, J., and Larkins, S. (1999). "Helping families move: relocation counseling for housing-voucher recipients." *Netherlands Journal of Housing and the Built Environment*, 14(1), 33–59. 25. Wates, N. (2013). *The Battle for Tolmers Square*: Taylor and Francis. 26. Wiesel, I., and Pawson, H. (2015). "Why do tenants leave social housing? Exploring residential and social mobility at the lowest rungs of Australia's socioeconomic ladder." *Australian Journal of Social Issues*, 50(4), 397–417. 27. Winter, A. K. (2016). ""Environmental sustainability? We don't have that here": Freetown Christiania as an unintentional eco-village." *ACME*, 15(1), 129–149.
Thematic/method misfit (n=66)
28. Abd Aziz, W. N. A. W., Hanif, N. R., and Ahmad, F. (2008). "The state intervention in achieving a quality urban living standard: a case study of rehabilitation of squatters' colony in Kuala Lumpur." *International Journal of Housing Markets and Analysis*, 1(4), 337–351. 29. Abdullah, Y. A., Kuek, J. N., Hamdan, H., and Zulkifli, L. M. (2017). "Combating squatters in Malaysia: do we have adequate policies as instrument?" *Planning Malaysia*(6), 25–36. 30. Addo, I. A. (2014). "Urban low-income housing development in Ghana: politics, policy and challenges", *Urban Planning: Practices, Challenges and Benefits*. Nova Science Publishers, Inc., pp. 89–118. 31. Adianto, J., Gabe, R. T., and Sihombing, A. (2022). "Houses with permeable walls: a case study from Kampong Kwitang, Central Jakarta." *International Journal of Design in Society*, 16(2), 1–18. 32. Akhtar, A. S., and Rashid, A. (2021). "Dispossession and the militarised developer state: financialisation and class power on the agrarian-urban frontier of Islamabad, Pakistan." *Third World Quarterly*, 42(8), 1866–1884. 33. Alam, S. M., and Markandey, K. (2020). *Consequences of Unplanned Growth: A Case Study of Metropolitan Hyderabad*. 34. Alkiser, Y., Dulgeroglu-Yuksel, Y., and Pulat-Gokmen, G. (2009). "An evaluation of urban transformation projects." *Archnet-Ijar International Journal of Architectural Research*, 3(1), 30–44. 35. Angelo, D. (2017). "Histories of a burnt house: an archaeology of negative spaces and dispossession." *American Anthropologist*, 119(2), 253–268. 36. Anierobi, C., and Obasi, C. O. (2021). "Urbanization and rural-urban migration: toward involving the church in addressing pro-poor urban housing challenges in Enugu, Nigeria." *SAGE Open*, 11(3). 37. Anindito, D. B., Indriansyah, N. R., Maula, F. K., and Akbar, R. (2019). "A quantitative perspective on Kampung Kota: elaborating definition and variables of Indonesian informal settlements: case study: Kelurahan Tamansari, Bandung City." *International Review for Spatial Planning and Sustainable Development*, 7(2), 53–74. 38. Aziz, F. A., Ujang, N., and Bakar, N. A. A. (2022). "Urban high-rise public housing for squatter resettlement: Desa Mentari as a case study." *New Design Ideas*, 6(2), 159–175. 39. Banerjee, B., and Verma, G. D. (1994). "Three Indian cases of upgradable plots." *Third World Planning Review*, 16(3), 263–275. 40. Bhuvaneswari, R. (2016). "‘Speculative spaces’: the material practices of urban entrepreneurialism", *Entrepreneurial Urbanism in India: The Politics of Spatial Restructuring and Local Contestation*. Springer Singapore, pp. 91–112. 41. Bikis, A., and Pandey, D. (2022). "Squatter settlement and informal urbanization: causes and consequences." *Environmental Science and Pollution Research*. 42. Bodur, A., and Dülgeroğlu Yüksel, Y. (2017). "Assessing change in quality of life following rehousing from slum settlements to social housing." *A/Z ITU Journal of the Faculty of Architecture*, 14(3), 53–65. 43. Brezar, V. (2012). "The future of urban dwelling design." *International Journal for Housing Science and Its Applications*, 36(2), 89–98. 44. Bykowa, E., Heldak, M., and Sishchuk, J. (2020). "Cadastral land value modelling based on zoning by prestige: a case study of a Resort Town." *Sustainability*, 12(19). 45. Cabannes, Y. (1997). "From community development to housing finance: from Mutiroesto Casa Melhorin Fortaleza, Brazil." *Environment & Urbanization*, 9(1), 31–58. 46. Čada, K. (2018). "‘They seemed like super businessmen’: financial Instruments in social housing policy." *Critical Housing Analysis*, 5(2), 56–67. 47. Cubukcu, E. (2011). "Which is better, social houses or gecekondus? An empirical study on Izmir’s residents." *Open House International*, 36(3), 97–107. 48. Davis, S. S. (1992). "An integrated sociophysical strategy for sustainable shelter development: a case study of slum and squatter settlements in Zambia." *Regional Development Dialogue*, 13(4), 118–137. 49. Dayaratne, R. (2010). "Creating sustainable habitats for the urban poor: redesigning slums into condominium high rises in Colombo", *Built Environment: Design, Management and Applications*. Nova Science Publishers, Inc., pp. 147–167. 50. Demoss-Norman, T. (2015). "From informal settlements to formality: a resettlement group's adaptation to a newly planned community in Port Elizabeth, South Africa." *Economic Anthropology*, 2(1), 224–240. 51. du Plessis, J. (2005). "The growing problem of forced evictions and the crucial importance of community-based, locally appropriate alternatives." *Environment and Urbanization*, 17(1), 123–134. 52. Eranil, M., and Gurel, M. O. (2022). "Social housing as paradoxical space: migrant women's spatial tactics inside Toki Uzundere Blocks." *Home Cultures*, 19(1), 23–48. 53. Erman, T. (2011). "Understanding the experiences of the politics of urbanization in two gecekondu (squatter) neighborhoods under two urban regimes: ethnography in the urban periphery of Ankara, Turkey." *Urban Anthropology*, 40(1–2), 67–108. 54. Erman, T. (2016). "Formalization by the state, re-informalization by the people: a gecekondu transformation housing estate as site of multiple discrepancies." *International Journal of Urban and Regional Research*, 40(2), 425–440. 55. Erman, T., and Hatiboğlu, B. (2017). "Rendering responsible, provoking desire: women and home in squatter/slum renewal projects in the Turkish context." *Gender, Place and Culture*, 24(9), 1283–1302. 56. Fang, Q., and Li, X. (2017). *Power versus Law in Modern China: Cities, Courts, and the Communist Party*, University Press of Kentucky. 57. Gbadegesin, J. T. (2022). "Does the COVID-19 affect tenants’ adherence to lease obligations in rental market? Property managers’ perspective." *Journal of Facilities Management*. 58. Ginsberg, Y. (1991). "Housing conditions of single-parent families in Israel." *Journal of Architectural & Planning Research*, 8(4), 307–319. 59. Giuliani, F., and Wiesenfeld, E. (2003). "Promoting sustainable communities: theory, research, and action." *Community, Work and Family*, 6(2), 159–181. 60. Glenn, J. M., Labossiere, R. P., and Wolfe, J. M. (1993). "Squatter regularisation: problems and prospects: a case study from Trinidad." *Third World Planning Review*, 15(3), 249–262. 61. Govender, V., and Loggia, C. (2021). "Adaptive reuse strategies in Durban inner city using hybrid mapping tools", in H. H. Magidimisha-Chipungu and L. Chipungu, (eds.), *Urban Inclusivity in Southern Africa*. pp. 219–250. 62. Govindasamy, A. (2010). "Indians and rural displacement: exclusion from region building in Malaysia." *Asian Journal of Political Science*, 18(1), 90–104. 63. Guan, Y. S. (2001). "Producing locality: space, houses and public culture in a Hindu festival in Malaysia." *Contributions to Indian Sociology*, 35(1), 33–64. 64. Guevara, T., and Wallace, J. (2022). "Urban land policy in San Carlos de Bariloche (2001–2019)(1) Contributions for a critical balance." *Urbano*, 25(45), 54–63. 65. Gür, E. A., and Yüksel, Y. D. (2019). "Analytical investigation of urban housing typologies in twentieth century Istanbul." *Archnet-IJAR*, 13(1), 93–111. 66. Hoffman, M., Pick, W. M., Cooper, D., and Myers, J. E. (1997). "Women's health status and use of health services in a rapidly growing peri-urban area of South Africa." *Social Science and Medicine*, 45(1), 149–157. 67. Hussain, R., Lobo, M. A., Inam, B., Khan, A., Qureshi, A. F., and Marsh, D. (1997). "Pneumonia perceptions and management: an ethnographic study in urban squatter settlements of Karachi, Pakistan." *Social Science and Medicine*, 45(7), 991–1004. 68. Itkin, E. (2022). "Post-destruction squatter phases in the Iron Age IIB–C Southern Levant." *Bulletin of ASOR*, 388, 51–72. 69. Jackson, A., and Archer, C. D. (2022). "Factors influencing Jamaican householders’ housing choice." *International Journal of Housing Markets and Analysis*, 15(5), 1053–1071. 70. Kaitilla, S. (1993). "Squatter settlements and residential mobility in the third world: the applicability of Turner's model." *International Journal for Housing Science and Its Applications*, 17(1), 031–048. 71. Koroglu, B. A., and Ercoskun, O. Y. (2006). "Urban transformation: a case study on 7 Cukurambar, Ankara." *Gazi University Journal of Science*, 19(3), 173–183. 72. Kruger, C. (2016). "(Dis)empowered whiteness: un-whitely spaces and the production of the good white home." *Anthropology Southern Africa*, 39(1), 46–57. 73. Kumar, C. S., Guruprasd, C., and Babu, P. N. (2013). "Urban slums community health conditions in India (a case study on Visakhapatnam city urban slums in Andhra Pradesh)." *International Journal of Social Science and Interdisciplinary Research*, 2(11). 74. Kundu, D. (2020). "Political and social inclusion and local democracy in Indian cities: case studies of Delhi and Bengaluru"Advances in *21st Century Human Settlements*. Springer, pp. 185–208. 75. La Grange, A., and Pretorius, F. (2005). "Shifts along the decommodification - commodification continuum: housing delivery and state accumulation in Hong Kong." *Urban Studies*, 42(13), 2471–2488. 76. Mukherjee, J. (2020). "Urban environmentalisms" *Exploring Urban Change in South Asia*. Springer, pp. 205–230. 77. Murad, M. W., and Siwar, C. (2007a). "Knowledge, attitude and behavior of the urban poor concerning solid waste management: a case study." *Journal of Applied Sciences*, 7(22), 3356–3367. 78. Murad, W., and Siwar, C. (2007b). "Waste management and recycling practices of the urban poor: a case study in Kuala Lumpur city, Malaysia." *Waste Management and Research*, 25(1), 3–13. 79. Naqvi, I. (2018). "Contesting access to power in urban Pakistan." *Urban Studies*, 55(6), 1242–1256. 80. nor Azriyati Wan Abd Aziz, W., Rosly Hanif, N., and Ahmad, F. (2008). "The state intervention in achieving a quality urban living standard: a case study of rehabilitation of squatters’ colony in Kuala Lumpur." *International Journal of Housing Markets and Analysis*, 1(4), 337–351. 81. Nwalusi, D. M., Okeke, F. O., Anierobi, C. M., Nnaemeka-Okeke, R. C., and Nwosu, K. I. (2022). "A study of the impact of rural-urban migration and urbanization on public housing delivery in Enugu Metropolis, Nigeria." *European Journal of Sustainable Development*, 11(3), 59–70. 82. Obeidat, B., Abed, A., and Gharaibeh, I. (2022). "Privacy as a motivating factor for spatial layout transformation in Jordanian public housing." *City, Territory and Architecture*, 9(1). 83. Obudho, R. A. (1992). "Urban and rural settlements in Kenya." *Regional Development Dialogue*, 13(4), 86–111. 84. Özdemirli, Y. K. (2014). "Alternative strategies for urban redevelopment: a case study in a squatter housing neighborhood of Ankara." *Cities*, 38, 37–46. 85. Qasim, M., and Zaidi, S. S.-U.-H. (2013). "Ensuring sustainable development through urban planning in Pakistan." *Mehran University Research Journal of Engineering and Technology*, 32(2), 207–220. 86. Ruiz-Tagle, J. (2016). "The persistence of segregation and inequality in socially diverse neighborhoods: a case study in La Florida, Santiago." *Eure*, 42(125), 81–108. 87. Severcan, Y. C. (2019). "Residential relocation and children's satisfaction with mass housing." *Metu Journal of the Faculty of Architecture*, 36(2), 61–84. 88. Shami, S. (1996). "Gender, domestic space, and urban upgrading: a case study from Amman." *Gender and Development*, 4(1), 17–23. 89. Sultana, A., Zeeshan, M., and Anzak, S. (2022). "A phenomenological analysis of rural women's childbirth preferences." *Sage Open*, 12(1). 90. Unsal, B. O. (2015). "State-led urban regeneration in Istanbul: power struggles between Interest groups and poor communities." *Housing Studies*, 30(8), 1299–1316. 91. Vries, P. d. (1995). "Squatters becoming beneficiaries: the trajectory of an integrated rural development programme." *European Review of Latin American and Caribbean Studies*(58), 45–70. 92. Wanie, C. M., Oben, E. E. E., Molombe, J. M., and Tassah, I. T. (2017). "Youth advocacy for efficient hostel management and affordable university students’ housing in Buea, Cameroon." *International Journal of Housing Markets and Analysis*, 10(1), 81–111. 93. Yildiz, H. T. (2006). "Change, continuity and home: the tent, traditional dwelling and squatter house in Turkey." *Open House International*, 31(4), 40–48.

**Box 2. All included references**

**Table:** 

Systematic searches (n=27)
1. Adebowale, O., and Simpeh, F. (2021). "Exploring the effects of studentification on neighbourhoods in Nigeria." *Journal of Facilities Management*. 2. Ahmed, S., and Salam, M. (2022). "Rental housing policies and associated legal covers: case of middle income formal housing in Karachi." *Journal of Urban Management*, 11(4), 488–499. 3. Akaabre, P. B., Poku-Boansi, M., and Adarkwa, K. K. (2018). "The growing activities of informal rental agents in the urban housing market of Kumasi, Ghana." *Cities*, 83, 34–43. 4. Ansah, J. W., Takyiakwaa, D., Atakora, E., and Amoah, M. (2020). "‘House to let’: housing agents, social networks and Ghana’s housing law and policy." *International Journal of Housing Policy*, 20(3), 390–416. 5. Awunyo-Akaba, Y., Awunyo-Akaba, J., Gyapong, M., Senah, K., Konradsen, F., and Rheinlander, T. (2016). "Sanitation investments in Ghana: an ethnographic investigation of the role of tenure security, land ownership and livelihoods." *BMC Public Health*, 16(594), (18 July 2016)-(18 July 2016). 6. Aykaç, G. (2022). "Muhtars becoming real estate agents: changing roles of neighborhood representatives in relation to the state-led urban transformation in Çinçin, Ankara, Turkey." *Journal of Urban Affairs*. 7. Chattaraj, D., Choudhury, K., and Joshi, M. (2017). "The Tenth Delhi: economy, politics and space in the post-liberalisation metropolis." *Decision*, 44(2), 147–160. 8. Ebekozien, A., Abdul-Aziz, A. R., and Jaafar, M. (2021). "Low-cost housing policies and squatters struggles in Nigeria: the Nigerian perspective on possible solutions." *International Journal of Construction Management*, 21(11), 1088–1098. 9. Ezeanah, U. (2021). "Quality housing: perception and insights of people in Benin City, Nigeria." *Urban Forum*, 32(1), 87–110. 10. Gbadegesin, J. T., Komolafe, M. O., Gbadegesin, T. F., and Omotoso, K. O. (2021). "Off-campus student housing satisfaction indicators and the drivers: from student perspectives to policy re-awakening in governance." *Journal of Human Behavior in the Social Environment*, 31(7), 889–915. 11. Grant, M. (1995). "Movement patterns and the medium-sized city. Tenants on the move in Gweru, Zimbabwe." *Habitat international*, 19(3), 357–69. 12. Gunter, A. (2014). "Renting shacks: landlords and tenants in the informal housing sector in Johannesburg South Africa." *Urbani Izziv*, 25(Special Issue), S96–S107. 13. Huchzermeyer, M. (2007). "Tenement city: the emergence of multi-storey districts through large-scale private landlordism in Nairobi." *International Journal of Urban and Regional Research*, 31(4), 714–732. 14. Huchzermeyer, M. (2008). "Slum upgrading in Nairobi within the housing and basic services market: a housing rights concern." *Journal of Asian and African Studies*, 43(1), 19–39. 15. Ikejiofor, U. (1997). "The private sector and urban housing production process in Nigeria: a study of small-scale landlords in Abuja." *Habitat International*, 21(4), 409–425. 16. Kamruzzaman, M. (2012). *Housing for the urban poor through informal providers, Dhaka, Bangladesh*. 17. Lategan, L., and Cilliers, J. (2019). "Informal backyard rentals through a social sustainability lens - a case study in Oudtshoorn, South Africa." *Town and Regional Planning*, 74, 64–78. 18. Lategan, L. G., and Cilliers, E. J. (2014). "The value of public green spaces and the effects of South Africa’s informal backyard rental sector", in S. Bastianoni, R. Pulselli, N. Marchettini, and C. A. Brebbia, (eds.), *The Sustainable City IX. Urban Regeneration and Sustainability*. WITPress, pp. 427–438. 19. Malik, S., Roosli, R., and Tariq, F. (2020). "Investigation of informal housing challenges and issues: experiences from slum and squatter of Lahore." *Journal of Housing and the Built Environment*, 35(1), 143–170. 20. Malpezzi, S., Tipple, A. G., and Willis, K. G. (1990). "Costs and benefits of rent control: a case study in Kumasi, Ghana." *World Bank Discussion Papers*, 74. 21. McKay, T., Fakudze, N., and Gunter, A. (2022). "The middle remains missing: class exclusion from the urban rental market in suburban Johannesburg." *Acta Academica*, 54(1), 113–133. 22. Mekonen, E. K. (2022). "Drivers of rising residential house rent in Wolkite town, Gurage zone, Ethiopia." *Cogent Social Sciences*, 8(1). 23. Miller, A. W., Agbenyo, F., and Mabakeng Menare, R. (2021). "Tenant management under COVID-19 pandemic season among informal settlement rental housing in Ghana." *Housing, Care and Support*, 24(1), 26–38. 24. Mwau, B., and Sverdlik, A. (2020). "High rises and low-quality shelter: rental housing dynamics in Mathare Valley, Nairobi." *Environment and Urbanization*, 32(2), 481–502. 25. Omeraki Çekirdekci, Ş. (2020). "There is no place like home: poverty and the squatter house." *Journal of Consumer Behaviour*, 19(3), 252–263. 26. Parnell, S., and Beavon, K. (1996). "Urban land restitution in post-apartheid South Africa: questions from the Johannesburg inner-city." *GeoJournal*, 39(1), 13–19. 27. Yung, B., and Lee, F. P. (2014). "‘Equal right to housing’ in Hong Kong housing policy: perspectives from disadvantaged groups." *Journal of Housing and the Built Environment*, 29(4), 563–582.
Manual searches (n=35)
28. Adu-Gyamfi, A., Poku-Boansi, M., and Cobbinah, P. B. (2020). "Homeownership aspirations: drawing on the experiences of renters and landlords in a deregulated private rental sector." *International Journal of Housing Policy*, 20(3), 417–446. 29. Arifin, L. S., and Dale, R. (2005). "Housing needs of migrant women industrial workers in Surabaya: insight from a life story approach." *Habitat International*, 29(2), 215–226. 30. Arku, G., Luginaah, I., and Mkandawire, P. (2012). "“You either pay more Advance Rent or you move out”: landlords/ladies’ and tenants’ dilemmas in the low-income housing market in Accra, Ghana." *Urban Studies*, 49(14), 3177–3193. 31. Asante, L. A., and Ehwi, R. J. (2022). "Housing transformation, rent gap and gentrification in Ghana’s traditional houses: insight from compound houses in Bantama, Kumasi." *Housing Studies*, 37(4), 578–604. 32. Cadstedt, J. (2010). "Private rental housing in Tanzania — a private matter?" *Habitat International*, 34(1), 46–52. 33. Contreras, Y., Neville, L., and González, R. (2019). "In-formality in access to housing for Latin American migrants: a case study of an intermediate Chilean city." *International Journal of Housing Policy*, 19(3), 411–435. 34. Datta, K. (1995). "Strategies for urban survival? Women landlords in Gaborone, Botswana." *Habitat International*, 19(1), 1–12. 35. Eduful, A. K., and Hooper, M. (2019). "Urban migration and housing during resource booms: the case of Sekondi-Takoradi, Ghana." *Habitat International*, 93, 102029. 36. Feather, C. (2018). "Between homeownership and rental housing: exploring the potential for hybrid tenure solutions." *International Journal of Housing Policy*, 18(4), 595–606. 37. Gilbert, A., and Varley, A. (1990). "The Mexican landlord: rental housing in Guadalajara and Puebla." *Urban Studies*, 27(1), 23–44. 38. Grant, M. (1996). "Vulnerability and privilege: transitions in the supply pattern of rental shelter in a mid-sized Zimbabwean city." *Geoforum*, 27(2), 247–260. 39. Hooper, M., and Cadstedt, J. (2014). "Moving beyond ‘community’ participation: perceptions of renting and the dynamics of participation around urban development in Dar es Salaam, Tanzania." *International Planning Studies*, 19(1), 25–44. 40. Huang, Y., and Yi, C. (2015). "Invisible migrant enclaves in Chinese cities: underground living in Beijing, China." *Urban Studies*, 52(15), 2948–2973. 41. Huq-Hussain, S. (1996). "Female migrants in an urban setting — the dimensions of spatial/physical adaptation: the case of Dhaka." *Habitat International*, 20(1), 93–107. 42. Jiang, Y., Waley, P., and Gonzalez, S. (2018). "‘Nice apartments, no jobs’: how former villagers experienced displacement and resettlement in the western suburbs of Shanghai." *Urban Studies*, 55(14), 3202–3217. 43. Kumar, S. (1996). "Landlordism in Third World urban low-income settlements: a case for further research." *Urban Studies*, 33(4–5), 753–782. 44. Kumar, S. (2001). "Embedded tenures: private renting and housing policy in Surat, India." *Housing Studies*, 16(4), 425–442. 45. Lancione, M. (2019). "The politics of embodied urban precarity: Roma people and the fight for housing in Bucharest, Romania." *Geoforum*, 101, 182–191. 46. Lemanski, C. (2009). "Augmented informality: South Africa's backyard dwellings as a by-product of formal housing policies." *Habitat International*, 33(4), 472–484. 47. Lin, S., and Li, Z. (2017). "Residential satisfaction of migrants in Wenzhou, an ‘ordinary city’ of China." *Habitat International*, 66, 76–85. 48. Ling, M. (2021). "Container housing: formal informality and deterritorialised home-making amid bulldozer urbanism in Shanghai." *Urban Studies*, 58(6), 1141–1157. 49. Lonardoni, F., and Bolay, J. C. (2016). "Rental housing and the urban poor: understanding the growth and production of rental housing in Brazilian favelas." *International Journal of Urban Sustainable Development*, 8(1), 49–67. 50. Łuczak, P., and Ławrynowicz, M. (2021). "How did the great transformation shape housing pathways? The case of older women living alone." *Housing Studies*, 1–18. 51. Lux, M., and Mikeszova, M. (2012). "Property restitution and private rental housing in transition: the case of the Czech Republic." *Housing Studies*, 27(1), 77–96. 52. Morris, A. (1999). "Tenant-landlord relations, the anti-apartheid struggle and physical decline in Hillbrow, an inner-city neighbourhood in Johannesburg." *Urban Studies*, 36(3), 509–526. 53. Mubiru, M. B., Nuhu, S., Kombe, W., and Limbumba, T. M. (2022). "Women-headed households and housing location preferences in the informal settlements: what can we learn from Luzira, Uganda?" *Habitat International*, 127, 102648. 54. Mukherji, A. (2015). "From tenants to homeowners: housing renters after disaster in Bhuj, India." *Housing Studies*, 30(7), 1135–1157. 55. Osmonova, K. (2016). "Experiencing liminality: housing, renting and informal tenants in Astana." *Central Asian Survey*, 35(2), 237–256. 56. Owusu-Ansah, A., Ohemeng-Mensah, D., Abdulai, R. T., and Obeng-Odoom, F. (2018). "Public choice theory and rental housing: an examination of rental housing contracts in Ghana." *Housing Studies*, 33(6), 938–959. 57. Ronald, R., and Jin, M. (2015). "Rental market restructuring in South Korea: the decline of the Chonsei sector and its implications." *Housing Studies*, 30(3), 413–432. 58. Sanyal, R. (2017). "A no-camp policy: interrogating informal settlements in Lebanon." *Geoforum*, 84, 117–125. 59. Sheng, Y. K., and Shrestha, M. (1998). "The development of housing for women factory workers in Bangkok: a case study of Klong Luang district." *Habitat International*, 22(3), 313–326. 60. Wells, J., Sinda, S. H., and Haddar, F. (1998). "Housing and building materials in low-income settlements in Dar es Salaam." *Habitat International*, 22(4), 397–409. 61. Wu, F. (2016). "Housing in Chinese Urban Villages: the dwellers, conditions and tenancy informality." *Housing Studies*, 31(7), 852–870. 62. Zhang, S., and Zheng, G. (2019). "Gating or de-gating? The rise of the gated village in Beijing." *Habitat International*, 85, 1–13.
Additions in targeted geographical regions (n=15)
63. Asante, L. A., Ehwi, R. J., and Gavu, E. K. (2022). "Advance rent mobilisation strategies of graduate renters in Ghana: a submarket of the private rental housing market." *Journal of Housing and the Built Environment*, 37(4), 1901–1921. 64. Asante, L. A., Gavu, E. K., Quansah, D. P. O., and Osei Tutu, D. (2018). "The difficult combination of renting and building a house in urban Ghana: analysing the perception of low and middle income earners in Accra." *GeoJournal*, 83(6), 1223–1237. 65. Erdõsi, S., Hegedüs, J., and Somogyi, E. (2000). "Is private rental an option for urban housing provision in Hungary?" *Journal of Housing and Built Environment*, 15(3), 267–291. 66. Gough, K. V., and Yankson, P. (2011). "A neglected aspect of the housing market: the caretakers of peri-urban Accra, Ghana." *Urban Studies*, 48(4), 793–810. 67. Hegedüs, J., Horváth, V., and Tosics, N. (2014). "Economic and legal conflicts between landlords and tenants in the Hungarian private rental sector." *International Journal of Housing Policy*, 14(2), 141–163. 68. Hegedüs, J., Lux, M., and Horváth, V. (2018). "Private Rental Housing in Transition Countries: An Alternative to Owner Occupation?". City: Palgrave Macmillan London. 69. Kupka, P., Walach, V., and Brendzová, A. (2021). "The poverty business: landlords, illicit practices and reproduction of disadvantaged neighbourhoods in Czechia." *Trends in Organized Crime*, 24(2), 227–245. 70. Lata, L. N. (2020). "Neoliberal urbanity and the Right to Housing of the urban poor in Dhaka, Bangladesh." *Environment and Urbanization ASIA*, 11(2), 218–230. 71. Luginaah, I., Arku, G., and Baiden, P. (2010). "Housing and health in Ghana: the psychosocial impacts of renting a home." *Int J Environ Res Public Health*, 7(2), 528–45. 72. Naik, M. (2015). "Informal rental housing typologies and experiences of low-income migrant renters in Gurgaon, India." *Environment and Urbanization ASIA*, 6(2), 154–175. 73. Owusu, G., Agyei-Mensah, S., and Lund, R. (2008). "Slums of hope and slums of despair: mobility and livelihoods in Nima, Accra." *Norsk Geografisk Tidsskrift - Norwegian Journal of Geography*, 62(3), 180–190. 74. Sendi, R., and Mali, B. Č. (2015). "Surviving in limbo: an insight into Slovenia's informal private rented housing sector." *Theoretical and Empirical Researches in Urban Management*, 10(4), 19–39. 75. Smith, S. (2017). "Landlordism and landlord–tenant relations in Kisumu and Kitale’s low-income settlements." *International Journal of Urban Sustainable Development*, 9(1), 46–63. 76. Uleme, C. M. (2021). *Slum Upgrading and the Rental Housing Sector: A study of landlord-tenant relationship in a Lagos (Nigeria) slum*. 77. Walach, V., Kupka, P., and Brendzová, A. (2021). "“The landlord treads on them, so everything’s fine”: exploitation and forced mobility in substandard private rental housing in Czechia." *Czech and Slovak Social Work*, 132–148.

## Data Availability

No data are associated with this article. Zenodo: *Systematic Review: PRISMA Checklist and Flow Diagram*. University of Bucharest. Zenodo. https://doi.org/10.5281/zenodo.12794284) ( Soaita, (2024)). PRISMA2024_checklist_sections.doc PRISMA_flowchart.docx Data are available under the terms of the Creative Commons Zero "No rights reserved" data waiver (CC0 4.0 Public domain dedication).
