# Supplementary material for: Systematic review: locating qualitative academic publications for reviewing tenants’ and landlords’ renting experiences and interaction in the Majority World
Source: Open Res Eur. 2024 Oct 2;4:178. Originally published 2024 Aug 13. [Version 2] doi: 10.12688/openreseurope.18234.2 (PMC11445643; doi:10.12688/openreseurope.18234.2)
Supplement: Peer review history

# Reviewer report: Reviewer response for version 2

**DOI:** 10.21956/openreseurope.20054.r48831

**Published:** 2025-01-20

**Authors:** Efa Tadesse Debele

**Affiliations:** 1 Samara University, Semera, Afar, Ethiopia

The article is fascinating. If the author included some articles from low-income countries or Africa like Private Rental Housing Market in Ethiopia, which was published by Heliyon, the overall article standard and scientific soundness would be critical. The article conveys very important scientific ideas and the potential to be replicated.

 Major Points

**Strengths:**

- **Focus:** The article addresses urgent and troubling issues in the private renting sector.
- **Methodology:** Methodological protocols for rental housing research are thoroughly investigated.
- **Relevance:** Emphasis on tenants' and landlords' experiences and interactions.
- **Methodological Rigor:** Employed meticulous methodological approaches.
- **Scientific Soundness:** The article maintains a high standard and conveys important scientific ideas.

**Criticism:**

- **Scope Limitation:** The article could have included more information on the private rental housing market in low-income countries in Africa, where the housing market's institutionalization is in danger.

Minor Points

- **Notability:** The author’s decision to open the methodology for scrutinization, replication, and adjustment depending on the needs and purposes of researchers in the area is commendable.
- **Replication Potential:** The article has the potential to be replicated due to its comprehensive methodological approach.

Overall, the article is a robust and well-constructed article, though it would benefit from a broader geographic focus. Great work in highlighting its strengths while suggesting areas for improvement!

Are sufficient details of the methods and analysis provided to allow replication by others?

Yes

Are the rationale for, and objectives of, the Systematic Review clearly stated?

Yes

Is the statistical analysis and its interpretation appropriate?

Yes

Are the conclusions drawn adequately supported by the results presented in the review?

Yes

Reviewer Expertise:

Social Problems, Socioeconomic, Political economy, Urban Sociology, Housing, Urban development, Gender development, Social dynamics and other aspects of Society.

I confirm that I have read this submission and believe that I have an appropriate level of expertise to confirm that it is of an acceptable scientific standard.

# Reviewer report: Reviewer response for version 2

**DOI:** 10.21956/openreseurope.20054.r48823

**Published:** 2025-01-06

**Authors:** Edward Yiu

**Affiliations:** 1 The University of Auckland, Auckland, Auckland, New Zealand

This article presents the development of a Critical Interpretative Synthesis (CIS) as a methodological framework for retrieving relevant literature from various databases, exemplified by qualitative studies on private rental housing in the Majority World. It makes a contribution by advancing a Findable, Accessible, Interoperable, and Reusable (FAIR) approach to literature search. It does not contain any statistical analysis, and thus the question on statistical analysis in the peer review form is irrelevant. It is noted that Version 1 of the manuscript has been reviewed by three scholars, and the author has addressed most of their feedback. This review report pertains to the revised version 2 of the manuscript. I found the author's responses to the reviewers’ comments satisfactory, and the manuscript is well-written.

 I offer the following suggestions for further improvement:

1. **Explanation of Less Common Keywords**: Providing remarks or legends to clarify the rationale behind the selection of less common keywords (e.g., *hmo*, *rooming*, *hous*) in the SCOPUS and Web of Science Boolean strings would enhance transparency and help readers better understand these decisions.
2. **Comparison Table of Search Results**: Including a table that compares search results across SCOPUS, Web of Science, and manual searches would be beneficial. This table could detail:
  - The total number of references retrieved from each source.
  - Duplicated references identified across databases.
  - Misfit references, along with reasons for their exclusion.
    Such a table would effectively visualize the impacts and contributions of different databases and search strategies.

These additions would improve the manuscript’s clarity and utility for other researchers replicating or adapting the approach.

Are sufficient details of the methods and analysis provided to allow replication by others?

Yes

Are the rationale for, and objectives of, the Systematic Review clearly stated?

Yes

Is the statistical analysis and its interpretation appropriate?

No

Are the conclusions drawn adequately supported by the results presented in the review?

Yes

Reviewer Expertise:

Real Estate Economics, Urban Studies

I confirm that I have read this submission and believe that I have an appropriate level of expertise to confirm that it is of an acceptable scientific standard, however I have significant reservations, as outlined above.

# Reviewer report: Reviewer response for version 2

**DOI:** 10.21956/openreseurope.20054.r44812

**Published:** 2024-10-10

**Authors:** Konstantin A Kholodilin

**Affiliations:** 1 DIW Berlin, Berlin, Germany

I am fully satisfied with the revision.

Are sufficient details of the methods and analysis provided to allow replication by others?

Yes

Are the rationale for, and objectives of, the Systematic Review clearly stated?

Yes

Is the statistical analysis and its interpretation appropriate?

Yes

Are the conclusions drawn adequately supported by the results presented in the review?

Yes

Reviewer Expertise:

Housing market; governmental regulations; economic history; business cycle analysis.

I confirm that I have read this submission and believe that I have an appropriate level of expertise to confirm that it is of an acceptable scientific standard.

# Reviewer report: Reviewer response for version 1

**DOI:** 10.21956/openreseurope.19705.r43559

**Published:** 2024-10-01

**Authors:** Danielle Jane Kerrigan

**Affiliations:** 1 Simon Fraser University, Burnaby, British Columbia, Canada

Echoing the other two reviewers, the choice of limiting the review to English language journal articles should be discussed and justified.

 Further discussion of how the ‘manual searches’ and ‘further additions’ differed would be appreciated specifically regarding peer recommendations.

 Overall, this systematic review is high quality and makes important contributions.

Are sufficient details of the methods and analysis provided to allow replication by others?

Yes

Are the rationale for, and objectives of, the Systematic Review clearly stated?

Yes

Is the statistical analysis and its interpretation appropriate?

Yes

Are the conclusions drawn adequately supported by the results presented in the review?

Yes

Reviewer Expertise:

private rental social relations, landlords and tenants, qualitative methodologies

I confirm that I have read this submission and believe that I have an appropriate level of expertise to confirm that it is of an acceptable scientific standard.

# Response

**Published:** 2024-10-01

**Authors:** Adriana Mihaela Soaita

**Affiliations:** Sociology, University of Bucharest, Bucharest, Bucharest, Romania

**Dear Dr** **Danielle Jane Kerrigan** Thank you for offering your time and valued expertise for peer reviewing in the Open Access Europe’s approach as well as for finding my contribution to be of high quality. Following the previous comments that I have already received, I added in version 2 of this paper the following. In response to the exclusive use of English, the following text was added:

- **Under “Preliminary decisions”:** (1) “… the limited resources of this AFFECTIVE-PRS’ work package of one-person/four-months…”; (2) Language: As a common practice (Garnham and Rolfe 2019; Lee et al. 2015; Rolfe et al. 2023; Soaita et al. 2020), searches were exclusive to publications written in English (for an exceptional multi-language review of policy documents see Kholodilin 2020). Given that the type of reference was restricted to academic publications as a proxy for quality, excluding other languages than English is not particularly problematic as it was shown that non-native English scholars prefer to publish in English for higher international visibility and greater potential for citations (e.g. Di Bitetti and Ferreras 2017).
- **Under “Summary”, in explaining Figure 2**: (3) …many countries remained unrepresented in the sample, a possible outcome of the exclusive use of English keywords. (4) These observations underscore the need for further research into the renting arrangements and experiences in the Majority World but also the usefulness of a systematic review of multilingual resources. While English remains the most spoken language in the world, particularly so in published scholarship, inclusion of Mandarin, Hindu, Spanish, Bengali and Russian language capabilities would increase the number of publications to be reviewed and, depending on the review’s guiding question, might enrich or validate its findings.

In response to the fact that the systematic searches returned fewer references than the manual ones, I added the following explanatory text:

- **Under “Piloting the keywords”**: The keywords for String 1 (see Table 1) were piloted separately in SCOPUS on January 9, 2023 in order to monitor the size of hits returned. For instance, a search for tenant* AND landlord* (i.e. publications that contains both words, either in the singular or in the plural, in their title, abstract and keywords) returned 1,578 hits, an unmanageable amount for the resources of the project (for more details see Soaita 2023).
- **Under “Manual searches”**: The apparently unusual fact that systematic searches returned 27 relevant references compared to the 35 returned by manual searches reflects a series of decisions that I have strategically taken to align the scope of the review with the available resources as well as the different searching options offered by databases (sophisticated Boolean combinations) vs journals (simple words). They refer to: my consideration of only two databases, which is a relatively rare approach in systematic reviewing and the tightening of the results via the three searching strings as opposed to the broad keywords used in the manual searches in journals. Had I used similarly broad keywords in database searches, the large number of overall returns would have made this project unfeasible while it would have discovered a larger set of relevant references. There is therefore a trade-off between broad and narrow database searches and the particular combination between databases and journal searches, which must be negotiated separately for each project. 

# Reviewer report: Reviewer response for version 1

**DOI:** 10.21956/openreseurope.19705.r43560

**Published:** 2024-09-04

**Authors:** Steve Rolfe

**Affiliations:** 1 University of Stirling, Stirling, Scotland, UK

This note provides a valuable guide to literature searching for a systematic review. I'm sure it will be useful for researchers conducting such reviews, particularly in related fields.

 I would suggest that there are a couple of issues which the author should address in order to strengthen the article further.

 Firstly, I thought it was interesting that the systematic application of Boolean search strings to academic databases actually produced fewer relevant items than the 'manual search' approach of examining specific journals and employing Google Scholar. I would suggest that the author should provide at least some comment on this outcome and the potential implications for search strategy.

 Secondly, as the previous reviewer has already noted, I think some further discussion of the exclusion of studies in languages other than English is necessary, particularly given the focus on Majority World countries.

 Finally, on page 3 there is a reference to 'less developed' countries and on page 5 a reference to the 'developed world'. I would recommend against using this language, especially as most of the paper refers to Majority/Minority World. If it is necessary to explain these terms, a wording related to income levels would be preferable to more/less developed.

Are sufficient details of the methods and analysis provided to allow replication by others?

Yes

Are the rationale for, and objectives of, the Systematic Review clearly stated?

Yes

Is the statistical analysis and its interpretation appropriate?

Yes

Are the conclusions drawn adequately supported by the results presented in the review?

Yes

Reviewer Expertise:

Social policy, housing, evaluation and review methodology.

I confirm that I have read this submission and believe that I have an appropriate level of expertise to confirm that it is of an acceptable scientific standard.

# Response

**Published:** 2024-09-08

**Authors:** Adriana Mihaela Soaita

**Affiliations:** Sociology, University of Bucharest, Bucharest, Bucharest, Romania

Dear Dr Rolfe S Thank you for offering your time and valued expertise for peer reviewing in the Open Access Europe’s approach as well as for finding my paper useful for helping researchers to conduct their literature reviews. Following your comments, I added: **In response to the fact that the systematic searches returned fewer references than the manual ones, I added the following explanatory text**:

- **Under “Piloting the keywords”**: The keywords for String 1 (see Table 1) were piloted separately in SCOPUS on January 9, 2023 in order to monitor the size of hits returned. For instance, a search for tenant* AND landlord* (i.e. publications that contains both words, either in the singular or in the plural, in their title, abstract and keywords) returned 1,578 hits, an unmanageable amount for the resources of the project (for more details see Soaita 2023).
- **Under “Manual searches”**: The apparently unusual fact that systematic searches returned 27 relevant references compared to the 35 returned by manual searches reflects a series of decisions that I have strategically taken to align the scope of the review with the available resources as well as the different searching options offered by databases (sophisticated Boolean combinations) vs journals (simple words). They refer to: my consideration of only two databases, which is a relatively rare approach in systematic reviewing and the tightening of the results via the three searching strings as opposed to the broad keywords used in the manual searches in journals. Had I used similarly broad keywords in database searches, the large number of overall returns would have made this project unfeasible while it would have discovered a larger set of relevant references. There is therefore a trade-off between broad and narrow database searches and the particular combination between databases and journal searches, which must be negotiated separately for each project. 

 
**In response to the exclusive use of English, the following text was added**:

- **Under “Preliminary decisions”:** (1) “… the limited resources of this AFFECTIVE-PRS’ work package of one-person/four-months…”; (2) Language: As a common practice (Garnham and Rolfe 2019; Lee et al. 2015; Rolfe et al. 2023; Soaita et al. 2020), searches were exclusive to publications written in English (for an exceptional multi-language review of policy documents see Kholodilin 2020). Given that the type of reference was restricted to academic publications as a proxy for quality, excluding other languages than English is not particularly problematic as it was shown that non-native English scholars prefer to publish in English for higher international visibility and greater potential for citations (e.g. Di Bitetti and Ferreras 2017).
- **Under “Summary”, in explaining Figure 2**: (3) …many countries remained unrepresented in the sample, a possible outcome of the exclusive use of English keywords. (4) These observations underscore the need for further research into the renting arrangements and experiences in the Majority World but also the usefulness of a systematic review of multilingual resources. While English remains the most spoken language in the world, particularly so in published scholarship, inclusion of Mandarin, Hindu, Spanish, Bengali and Russian language capabilities would increase the number of publications to be reviewed and, depending on the review’s guiding question, might enrich or just validate its findings.

 
**To explain the terms Majority/Minority World the following text was introduced (also, all references to ‘developed’ states were replaced)**:

- …an expression devised by Alam (2008) to refer to a political-economic geography by what it has (the greatest majority of the global population), rather than by what it lacks (economic development), thus challenging the discursive political hegemony of its complement, the Minority World (see also Simone and Fauzan 2013)

# Reviewer report: Reviewer response for version 1

**DOI:** 10.21956/openreseurope.19705.r43558

**Published:** 2024-09-03

**Authors:** Konstantin A Kholodilin

**Affiliations:** 1 DIW Berlin, Berlin, Germany

I find that this note is a useful guide to a systematic literature search. It should help the researchers to conduct their literature reviews.

 Although the purpose of this note is purely technical, still it would be very interesting and useful for the readers, if the author could explain in a few words what "Affective Economies" is.

 A concise explanation of the FAIR approach is necessary.

 If I correctly understand the FAIR approach, the references must be easy to find. However, my experience with SCOPUS and Web of Science databases shows me that their use is not very easy and may require paying a fee which can be an insurmountable obstacle in particular for the researchers from the Global South. I hope that I am mistaken. But if it is true, what are the ways to overcome this challenge.

 Is exclusion of studies written in other languages than English is fruitful? It can lead to information losses, which are especially relevant when the countries beyond the Majority World are the focus of your study.

Are sufficient details of the methods and analysis provided to allow replication by others?

Yes

Are the rationale for, and objectives of, the Systematic Review clearly stated?

Yes

Is the statistical analysis and its interpretation appropriate?

Yes

Are the conclusions drawn adequately supported by the results presented in the review?

Yes

Reviewer Expertise:

economics, housing, history

I confirm that I have read this submission and believe that I have an appropriate level of expertise to confirm that it is of an acceptable scientific standard, however I have significant reservations, as outlined above.

# Response

**Published:** 2024-09-08

**Authors:** Adriana Mihaela Soaita

**Affiliations:** Sociology, University of Bucharest, Bucharest, Bucharest, Romania

**Dear Dr Kholodilin KA** Thank you for offering your time and valued expertise for peer reviewing in the Open Access Europe’s approach as well as for finding my paper useful for helping researchers to conduct their literature reviews. Following your comments, I added: **A brief explanation for the term “Affective economies” was given** **in the second paragraph of the introduction**:

- “As the project is centred on the notion of affective economies, a brief theoretical explanation is warranted. The concept was authored by Sarah Ahmed (2004) and has since attracted a small but growing subfield of studies. Starting from the widely agreed statement that “economies are social and material, as well as psychic” (p.121), Ahmed conceptualises emotions as being affective investments in social norms (not just psychological dispositions) that is collective affects which construct collective bodies, e.g. the nation, the racialized skin, the poor, the undeserving, the refugee or, as I suggest, certain bodies of tenants or landlords. The concept helps us see the economies of private renting as a zone imbricated with collective rather than individual emotions, which are shared between some people and opposed, rejected or unrecognized by other people. It can also help organise the growing but still cluttered focus on emotion in housing scholarship.”

**The following text was added as a concise explanation of the FAIR**:

- **In the “Introduction”:** …references FAIR—that is Findable, Accessible, Interoperable, and Reusable—for other academic users albeit and regrettably only for those privileged whose affiliated institutions offer entry beyond the pay-wall of academic journals and databases and who more often than not belong to the Minority World.
- **In the “Conclusion”:** This protocol aimed to enhance the AFFECTIVE-PRS reviewing approach by making it FAIR—that is Findable, Accessible, Interoperable, and Reusable for other academic users, albeit and regrettably only for those privileged whose affiliated institutions offer entry beyond the pay-wall of academic journals and databases and who more often than not belong to the Minority World. The fact that Minority World’s institutions increasingly finance open access publications and that more academic journals allow publication of pre-prints is encouraging and should be hastened but access to database searching remains exclusive to pay-walls. From this point of view, Open Research Europe is an important development. Nonetheless, in the current academic institutional landscape, conducting a systematic review mostly remains a privilege of the Minority World.

**In response to the exclusive use of English, the following text was added**:

- **Under “Preliminary decisions”:** (1) “… the limited resources of this AFFECTIVE-PRS’ work package of one-person/four-months…”; (2) Language: As a common practice (Garnham and Rolfe 2019; Lee et al. 2015; Rolfe et al. 2023; Soaita et al. 2020), searches were exclusive to publications written in English (for an exceptional multi-language review of policy documents see Kholodilin 2020). Given that the type of reference was restricted to academic publications as a proxy for quality, excluding other languages than English is not particularly problematic as it was shown that non-native English scholars prefer to publish in English for higher international visibility and greater potential for citations (e.g. Di Bitetti and Ferreras 2017).
- **Under “Summary”, in explaining Figure 2**: (3) …many countries remained unrepresented in the sample, a possible outcome of the exclusive use of English keywords. (4) These observations underscore the need for further research into the renting arrangements and experiences in the Majority World but also the usefulness of a systematic review of multilingual resources. While English remains the most spoken language in the world, particularly so in published scholarship, inclusion of Mandarin, Hindu, Spanish, Bengali and Russian language capabilities would increase the number of publications to be reviewed and, depending on the review’s guiding question, might enrich or validate its findings.
